# ’Carotid spells’ - transient focal neurological episodes associated with convexity subarachnoid haemorrhage in severe ipsilateral extracranial internal carotid artery stenosis: a case report and concise literature review

**DOI:** 10.1186/s12883-026-04751-6

**Published:** 2026-02-19

**Authors:** Jozsef Norbert Nemes, Emil Ferencz, Peter Klivenyi, Levente Szalardy

**Affiliations:** 1 Department of Neurology, Albert Szent-Györgyi Clinical Center, Albert Szent-Györgyi Medical School, University of Szeged, Semmelweis u. 6, Szeged, H-6725 Hungary; 2 HUN-REN-SZTE Neuroscience Research Group, Semmelweis u. 6., Szeged, H-6725 Hungary

**Keywords:** Atherosclerosis, Carotid artery, Convexity subarachnoid haemorrhage, Endarterectomy, Extracranial, Spell, Stenosis, Transient focal neurological episode

## Abstract

**Background:**

Convexity subarachnoid haemorrhage (cSAH) is most frequently caused by cerebral amyloid angiopathy (CAA), often presenting with transient focal neurological episodes (TFNEs, a.k.a. amyloid spells). In younger patients, posterior reversible encephalopathy, reversible cerebral vasoconstriction syndrome, and sinus thrombosis are the most frequent aetiologies. Reports on extracranial internal carotid artery (ICA) stenosis-associated cSAHs are exceptional.

**Case presentation:**

A 46-year-old female with a history of heavy smoking presented with repetitive stereotypical right-sided sensory TFNEs. An acute cranial computed tomography revealed multi-lobar left hemispheric cSAHs. The angiography demonstrated severe bilateral extracranial atherosclerotic ICA stenosis with left-sided predominance and no intracranial involvement. In addition to confirming the cSAHs, a magnetic resonance imaging revealed acute/subacute small ischaemic alterations in the left anterior watershed area. A detailed diagnostic work-up did not reveal an alternative aetiology. Based on the stereotyped presentation, in addition to repetitive transient ischaemic attacks secondary to stenosis, focal epileptic or spell-like episodes triggered by the cSAHs were considered as plausible mechanisms underlying the TFNEs. Given the high TFNE frequency on admission, empirical antiepileptics were initiated; the patient subsequently became asymptomatic and the TFNEs stopped. Secondary stroke prevention was initiated on day 9. Considering the asymptomatic state and the recent intracranial haemorrhages, vascular surgeons opted for elective endarterectomy of the left ICA, which was performed 6 weeks later. The contralateral endarterectomy was performed three months later. The patient is asymptomatic and the antiepileptics have been successfully tapered. We propose that, in our case, cSAHs could be attributed to severe ipsilateral ICA stenosis and consequently recruited fragile leptomeningeal collaterals, similarly to that seen in moyamoya disease/syndrome. The TFNEs most likely developed secondary to a cSAH-associated mechanism reminiscent of that seen in amyloid spells in CAA.

**Conclusions:**

cSAH is a rare but often symptomatic manifestation of an ipsilateral significant extracranial ICA stenosis, which widens the spectrum of its possible clinical manifestations. Expanding the literature of the few cases previously reported with a similar scenario, our case draws attention to the significance of extracranial vascular imaging in the setting of TFNEs and/or cSAHs, to promote the timely diagnosis of stenotic alterations of potential surgical relevance.

## Background

Spontaneous (i.e., non-traumatic) convexity subarachnoid haemorrhage (cSAH) represents approximately 5–10% of all SAHs, and is often underdiagnosed in the clinical setting [1, 2]. In cSAH, the sulcal haemorrhage by definition does not extend past the convexal surface, contrasting with SAH due to aneurysm rupture at predilection points of the circle of Willis arteries at the skull base [3]. Radiologically, cSAH can be visualised by computed tomography (CT) scan as focal curvilinear hyperdensity within a sulcus, whereas on magnetic resonance imaging (MRI), it presents as a sulcal hypointensity on susceptibility-weighted imaging (SWI) or gradient echo (GRE) sequences, with corresponding hyperintensity on the fluid-attenuated inversion recovery (FLAIR) sequence (a.k.a. sulcal non-nulling) [4].

The most frequent cause of non-traumatic cSAH in the elderly is cerebral amyloid angiopathy (CAA), where it often presents with typically stereotyped transient focal neurological episodes (TFNEs), a.k.a. ’amyloid spells’, considered to develop due to cortical spreading depression triggered by the haemorrhage [5]. cSAH in CAA is thought to represent the acute episodic manifestation of cortical superficial siderosis (cSS), a highly specific haemorrhagic feature in sporadic (and also in genetic [6]) CAA, which is associated with a high risk of developing symptomatic lobar intracerebral haemorrhage [7].

In younger patients, however, posterior reversible encephalopathy syndrome (PRES), reversible cerebral vasoconstriction syndrome (RCVS, including postpartum angiopathy and vascular changes related to amphetamine use), and cerebral venous thrombosis (CVT) are the most common underlying aetiologies of cSAH. These are generally characterised by severe headache (frequently described as worst ever), distinctive intracranial angiographic and/or parenchymal MRI alterations, and suggestive provoking factors [2, 8, 9]. In addition, other rare causes such as moyamoya disease and syndrome (associated with intracranial internal carotid artery (ICA) stenosis), endocarditic embolization, brain abscess, neoplasm, cerebral vasculitis (primary and secondary), and vascular malformations (including dural arteriovenous fistulas) have been described as predisposing conditions [2, 8, 10–13].

Here we report a young case with repetitive stereotyped left hemispheric TFNEs in association with multiple ipsilateral cSAHs and severe atherosclerosis with a subtotal occlusion of the ipsilateral extracranial ICA, successfully treated by antiepileptics and carotid endarterectomy (CEA). The CARE guidelines were followed [14].

## Case presentation

**Fig. 1:**
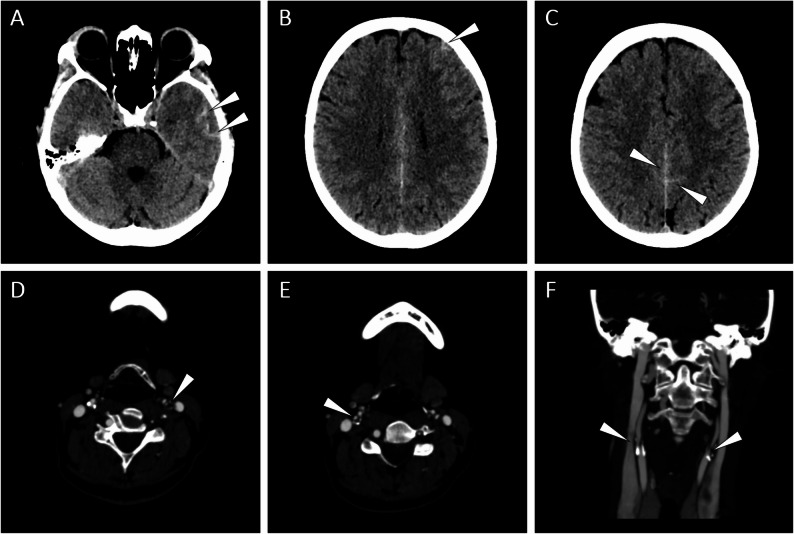
Acute cranial/carotid CT/CTA imaging. Axial non-contrast CT scans showing left temporal (**A**), left frontal (**B**), and left parafalcine cSAHs (**C**). Axial carotid CTA demonstrating subtotal occlusion of the ipsilateral ICA (**D**) and a significant stenosis of the contralateral ICA (**E**). Coronal CTA showing bilateral atherosclerotic plaques of the stenotic ICAs (**F**). cSAH, convexity subarachnoid haemorrhage; CT, computed tomography; CTA, CT angiography; ICA, internal carotid artery

**Fig. 2:**
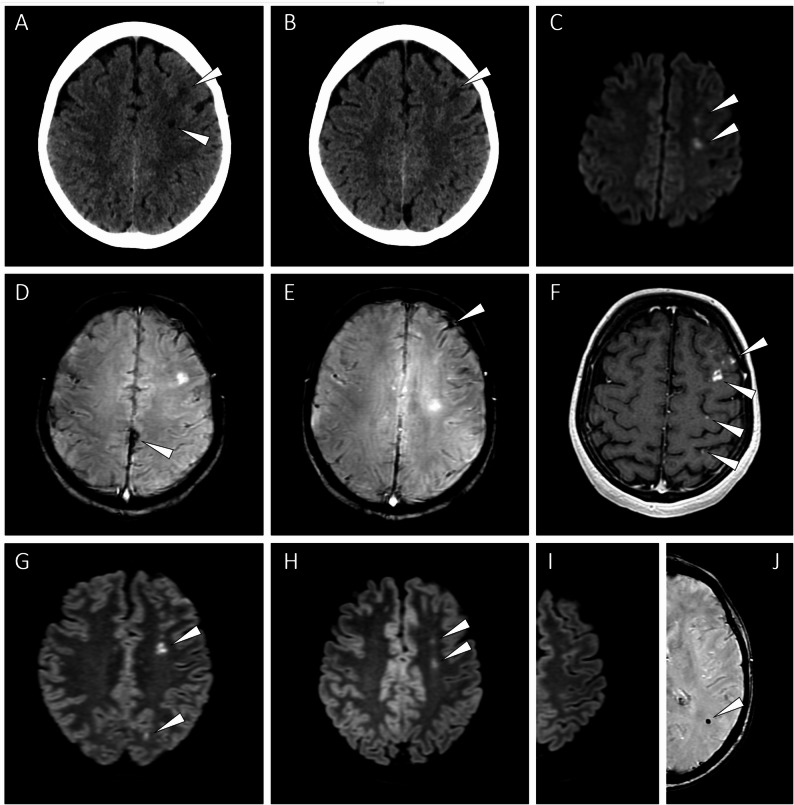
Follow-up CT and MRI scans. Subacute subcortical and cortical hypodensities in the left-sided anterior border zone, 2 days after presentation (**A**, **B**). Axial DWI MRI confirming acute ischaemia in the subcortical alterations of the left frontal lobe, 7 days after presentation (**C**). Representative images of axial SWI MRI confirming the cSAHs (**D**, **E**). Axial post-contrast T1 MRI showing luxury perfusion in the ischaemic lesions (**F**). Axial FLAIR MRI demonstrating hyperintense lesions consistent with vasogenic oedema (**G**). Follow-up FLAIR images showing minimal hyperintense lesions consistent with chronic infarcts confined to the subcortical alterations with initial DWI hyperintensity (**H**). No residual infarcts can be appreciated in the cortical luxury perfusion sites (**I**). A microbleed appeared in the immediately subcortical white matter of the left parietal lobe on SWI (**J**). cSAH, convexity subarachnoid haemorrhage; CT, computed tomography; DWI, diffusion-weighted imaging; FLAIR, fluid-attenuated inversion recovery; MRI, magnetic resonance imaging; SWI, susceptibility-weighted imaging

A 46-year-old female patient was admitted to the emergency room (ER) with repetitive, stereotyped, short-lasting (5-10-min) episodes of right-sided facial paraesthesias, which she experienced 5–10 times daily in the past 5 days. On the day of presentation, the transient symptom was also associated with right distal upper limb paraesthesia and headache. She measured systolic blood pressure values of ~ 160 mmHg on these days. She also admitted a recent transitory sight loss of the left eye during a neck extension, for which she did not seek medical attention. She denied head trauma. Her past medical history was relevant for a treated hypertension and heavy smoking (both actively and passively). The family history was relevant for several strokes in a grandmother. On admission, the neurological examination revealed right-sided facial tactile hypaesthesia and minimal pyramidal signs of the left upper limb (i.e., Hoffmann-Trömner sign and inverted radial reflex without asymmetry in deep tendon reflexes), without any other pathological findings. Urgent blood tests were unremarkable. An urgent cranial CT showed multiple cSAHs in the left temporal, parietal, and frontal lobes, without any hypodensities suspicious of aetiologies associated with vasogenic oedema (Fig. 1A-C). The carotid CT angiography excluded vascular malformations and the multifocal segmental narrowing and dilatation (‘string-of-beads’) pattern typical of RCVS, but revealed a severe bilateral extracranial ICA stenosis due to atherosclerotic plaques with left-sided predominance, where a subtotal occlusion was appreciated (Fig. 1D-F). A second cranial CT performed 24 h later showed no progression in the haemorrhages, but revealed novel subacute ischaemic lesions in the left-sided anterior border zone, both subcortically and cortically (Fig. 2A-B).

A comprehensive differential diagnostic work-up was initiated. The HDL cholesterol level was slightly decreased (0.96 mmol/L; 37.2 mg/dL), and the LDL level was above target range (2.34 mmol/L; 90.5 mg/dL). Apart from a borderline lipoprotein(a) level (29 mg/dL) and a heterozygous state for Factor V Leiden mutation, the thrombophilia screen was unrevealing. Autoimmune laboratory tests did not confirm a systemic autoimmune disorder, and a comprehensive panel of serological tests also ruled out cerebral small vessel vasculitis secondary to infection. Because the angiographic findings and the clinical presentation, including the absence of thunderclap headache, were not suggestive of RCVS, and the patient denied illicit drug use with no clinical indicators to suggest otherwise, formal toxicology screening was not performed. The cerebrospinal fluid (CSF) analysis showed changes largely corresponding to a traumatic puncture (7–13 leukocytes/µL, 4211–5120 erythrocytes/µL, albumin quotient 7.6*10^−3^), with the amount of haemoglobin degradation products being below the detection limit of routine spectrophotometry. Although the patient had no suggestive history of iatrogenic CAA (such as childhood neurosurgical procedure or human cadaveric growth hormone replacement [15]) or a dominantly inherited familial CAA, the CSF Alzheimer’s disease core biomarker panel was assessed to rule out early-onset CAA, and the results were within normal limits (amyloid-β_1−42_: 851.1 pg/ml, Tau: 259.4 pg/ml, phosphorylated Tau_181_: 47.5 pg/ml, ELISA, FUJIREBIO). This was in line with the lack of lobar cerebral microbleeds or chronic cSS on the subsequent MRI (detailed later). The echocardiography was normal, without alterations suspicious of endocarditis. To specifically address potential aetiologies underlying the cSAHs, a contrasted brain MRI was performed (Fig. 2C-G), which confirmed the subacute cSAHs and the left-sided anterior border zone subacute ischemic lesions, with diffusion-weighted imaging (DWI)-FLAIR match, and (especially for the cortical lesions) enhancement of the contrast agent for the latter (i.e., luxury perfusion). No other pathological findings were present to support alternative causes of cSAH, including PRES or CVT.

Based on these observations, we considered the multifocal left-sided cSAHs to be secondary to the chronic subtotal occlusion of left-sided extracranial ICA, arising from fragile pial-pial anastomotic collaterals between cortical branches of the middle and posterior cerebral arteries, recruited as compensatory pathways for chronic hypoperfusion, analogous to those that develop in moyamoya disease/syndrome secondary to intracranial ICA stenosis.

As the TFNEs raised the suspicion of a focal epileptic disorder and were repeated multiple times on the first day of admission, antiepileptic treatment was initiated with levetiracetam. No TFNEs occurred thereafter. An electroencephalography (EEG; already on levetiracetam) did not show interictal epileptic discharges.

The patient was referred to vascular surgery, which opted for a delayed CEA, scheduled 6 weeks later, taking into account the lack of severe neurological symptoms, the presence of intracranial bleedings, and the requirement of heparin use during the surgical procedure. During in-hospital care, the patient did not need any antihypertensives, and single antiplatelet therapy with aspirin was started on the 9th day (100 mg daily), with statin (atorvastatin 40 mg daily).

The CEA of the left ICA was performed 6 weeks later, the postoperative period was uneventful. One month later, she presented in the ER with an episode of right-sided facial drooping and faciobrachial sensory complaints accompanying a hypertensive surge; an urgent cranial/carotid CT angiography showed no signs of new haemorrhage or re-stenosis, and the complaints spontaneously resolved. At this point, the minimal left upper limb pyramidal signs present on first admission were no longer observable. We interpreted this as indicative of a hypoperfusion-related aetiology of these signs, partly attributable to the right-sided ICA stenosis and to the contralateral near-total occlusion present at first admission, which had together compromised the circle of Willis before the first surgery. Given this constellation, it is difficult to ascertain whether such a transient, hypoperfusion-mediated minimal symptom would have occurred if the right-sided ICA stenosis had been the sole lesion, and therefore to regard the right ICA stenosis as symptomatic by default. After first surgery, the NASCET-equivalent degree of the right ICA stenosis was 80%, caused by a mixed atheromatous and soft plaque. Carotid sonography recorded a 170 cm/s peak systolic velocity, corresponding to a 50–69% stenosis (noting that this was a preliminary/orienting ultrasonography performed by a vascular surgeon, not a certified radiologist). Given the history of the patient and the collateral vascular complications developed on the left-side, surgical treatment of the right-sided stenosis was decided by the vascular surgeons. Three months after the first surgery, the right-sided ICA endarterectomy was performed without complications. Since then, the patient is without complaints and any focal neurological signs. A follow-up MRI 6 months after baseline showed a single novel microbleed in the immediate subcortical white matter of the left parietal lobe, which we interpret as a possible parenchymal analogue of the cSAHs, potentially arising from a fragile cortical collateral. Only minimal residual FLAIR alterations remained on this MRI, affecting only a subset of sites that had previously demonstrated DWI hyperintensity or cortical contrast enhancement, indicating that not all prior lesions evolved into permanent infarcts. These findings also support the hypothesis that these alterations arose secondary to hypoperfusion in a watershed-like pattern, rather than embolization (Fig. 2H-J**)**.

## Discussion

Significant extracranial ICA stenosis is an extremely rare possible cause of cSAH; therefore, other aetiologies should be considered and ruled out before establishing the diagnosis, for which non-invasive approaches have been recommended [16]. The first consideration is age, where PRES, RCVS, and CVT are to be primarily considered in patients < 60 years and suspicious history, whereas CAA-related cSAHs are more typical of the elderly [2, 17, 18]. The distinguishing features include (1) symmetric biparietal vasogenic oedema and encephalopathy-like presentation associated with (pre)eclampsia or hypertensive crisis in PRES, (2) a thunderclap headache with angiographic beading in the intracerebral vasculature and not-uncommonly acute watershed infarcts in a postpartum scenario or amphetamine use in RCVS (which may as well be associated with PRES), and (3) a venous infarct and characteristic density/signal changes with non-opacification of the affected sinus(es) in contrast-enhanced imaging in CVT, frequently in association with oral contraceptives, inherited thrombophilia, or systemic malignancy [8]. The diagnosis of probable CAA can be established by the Boston criteria v2.0 using MRI, generally based on the presence of multiple strictly lobar and/or convexity/cortical superficial haemorrhages on SWI, without other cause in patients ≥ 50 years of age [19]. CAA-related inflammation (CAA-RI) can also present with bilateral posterior vasogenic oedema reminiscent of PRES in relatively younger patients (≥ 40 years), and both conditions can be associated with cSAH, which represents a diagnostic challenge; however, in CAA-RI, the oedema is typically asymmetric and the characteristic hypertensive crisis is missing [4]. More recently, iatrogenic CAA has emerged as a distinct entity in individuals with relevant neurosurgical or hormonal intervention in childhood, presenting with CAA-related haemorrhagic alterations (including cSAHs) and, occasionally, CAA-RI, two to three decades after iatrogenic transmission [15]. Additional less frequent causes of cSAHs include vascular malformations and embolization due to endocarditis, which can be addressed by angiography and echocardiography, respectively. Further rare but recognised causes of cSAHs include moyamoya disease/syndrome, characterised by progressive stenosis of the terminal intracranial ICA and the proximal MCA and PCA branches, with consequential recruitment of basal deep perforator (puff of smoke), pial-pial (leptomeningeal), and transdural (from the external carotid artery branches) collaterals. While moyamoya disease is idiopathic and has strong genetic predisposition, moyamoya syndrome develops secondary to aetiologies such as neurofibromatosis type 1, Down’s syndrome, sickle cell disease, irradiation vasculopathy, meningitis, cerebral tumours, and autoimmune disorders, with atherosclerosis being excluded from the list of secondary causes [20].

Atherosclerotic stenosis of intracranial and most recently extracranial ICA has emerged as an underlying aetiology of cSAHs to be considered in the differential diagnosis. Though compelling evidence is missing, there is a strong rationale to suggest that recruited fragile pial anastomoses could be the underlying source of haemorrhage in this scenario [12, 13]. Notably, while the recruitment of pial anastomoses in both extra- and intracranial ICA stenoses has been well established [21], the deep basal perforator collaterals pathognomonic of moyamoya disease/syndrome represent a proliferative neovascular response with distinctive histopathological features (a.k.a. moyamoya vessels), and by definition do not occur in atherosclerotic ICA stenosis, even when the intracranial terminal segments are affected [12, 22].

Our patient presented with stereotyped left hemispheric TFNEs in association with multiple left hemispheric cSAHs and severe bilateral extracranial atherosclerotic ICA stenosis with ipsilateral predominance. The history, the clinical picture, and the overall radiographic presentation were not suggestive of more typical causes of cSAHs, and the detailed work-up (including CSF Aβ_1−42_ levels) did not support an alternative aetiology. We are aware of a total of 13 other cases published in the literature with cSAHs associated with ipsilateral extracranial ICA stenosis without other cause or intracranial stenosis (Table 1) [13, 23–28]. In these patients, the mean age at presentation was 59.3, the female:male ratio was 1:1, 57.1% affected the right side. The ICA involvement on the corresponding side was total occlusion in 42.8%, severe stenosis or near-total occlusion in the rest. In cases with bilateral severe stenosis, the side of the cSAH corresponded to that of the more severe stenosis, similarly to our case [23], whereas there was bilateral occlusion in 1 case [28]. The reported symptomatic presentation included TFNEs (corresponding to cSAH locations) in 50.0%, amaurosis fugax in 21.4%, headache in 35.7%, and usually mild sensory or motor focal neurological deficits on examination in 57.1%. Definite ischaemic alterations were noted in the same 57.1% as those presenting with focal neurological deficits on examination. Manifest seizures were not reported, except for one patient with focal seizures considered secondary to hyperperfusion syndrome after CEA [26]. Standard secondary stroke prevention approaches were supplemented with CEA in all cases without total occlusion where therapy was reported (Table 1). The course was favourable in all cases with available follow-up.

**Table 1:** Reported cases of cSAH associated with isolated severe extracranial ICA stenosis/occlusion

Ref.	Sex	Age		cSAH location	Acute ischaemia location	Extent of ipsilateral stenosis	Presenting symptoms	Risk factors	Therapy	Outcome
[23]	M	28		L inf. Te	L MCA	tight	FND (R mild paresis and SD)	smoking, HC	CEA	NA
	F	50		L Fr	not noted	95%	headache, amaurosis fugax	smoking, DL	CEA	NA
	F	55		R inf. Te	R MCA	90%	FND (L mild paresis and SD), TFNEs (visual aura), headache	smoking, HC, HT	CEA	NA
[24]	M	70		L CS	no ischaemia	80%	TFNEs (aphasia, dysarthria, R face-arm SD)	HT	AC, AED, CEA	CR
[25]	F	46		L preCS	L MCA, L MCA/PCA BZ	occlusion	FND (dysarthria, dysphasia)	obesity, HT, DM, DL, smoking	APT (aspirin), statin	CR
	M	59		R CS	R MCA, R MCA/PCA BZ	occlusion	FND (neglect, R ocular deviation, L paresis)	DM, ischaemic cardiomyopathy, TIAs	APT (aspirin), statin	CR
[13]^#^	M	50		R postCS	R MCA	85%	TFNE (L facial palsy), FND (dysarthria)	NA	APT, statin, CEA	CR
	F	67		R Fr, Pa	R MCA	95%	FND (L face-arm paresis and SD), headache	NA	APT, statin, CEA	mRS 2
[26]	F	78		L Pa	L MCA (2 months later)	90%	FND (dysarthria, facial palsy), headache	DM	APT, CEA, AED^*^	CR
[27]	F	79		R CS	not noted	occlusion	TFNE (L arm paresis, dysphasia)	NA	NA	NA
[28]	M	79		R Fr	no ischaemia	CCA occlusion	R amaurosis fugax	AF	APT, statin	CR
	F	63		R Fr	not noted	occlusion	TFNE (L leg paresis)	HT, DL, smoking	APT, statin	CR
	M	60		R hem.	not noted	occlusion	TFNE (dysarthria, arm paresis)	DM, HT, smoking	APT, statin	CR
PR	F	46		L Te, Pa, Fr	L MCA/ACA BZ	near occlusion	TFNEs (R face-arm SD), L amaurosis fugax, FND (R face SD), headache	smoking, HT, heterozygous Leiden mutation	APT (aspirin), statin, AED, CEA	CR

*AC* anticoagulation, *ACA* anterior cerebral artery, *AED* antiepileptic drug, *AF* atrial fibrillation, *APT* antiplatelet therapy, *BZ* border zone, *CEA* carotid endarterectomy, *CR* complete recovery, *CS* central sulcus, *cSAH* convexity subarachnoid haemorrhage, *DAPT* dual antiplatelet therapy, *DL* dyslipidaemia, *DM* diabetes mellitus, *F* female, *FND* focal neurological deficit, *Fr* frontal, *HC* hypercholesterolaemia, *hem* hemisphere, *HT* hypertension, *ICA* internal carotid artery, *M* male, *MCA* middle cerebral artery, *NA* not available, *Pa* parietal, *PCA* posterior cerebral artery, *PR* present report, *Ref*. Reference, *SD* sensory disturbance, *Te* temporal, *TIA* transient ischaemic attack

^#^cases with intracranial stenosis or suspected duplicate reports were omitted

^*^only after postprocedural focal seizures

The aetiology of TFNEs in such a scenario is unclear. First of all, transient ischaemic attack (TIA, embolic or hypoperfusion-related) might be a plausible mechanism due to the underlying ICA stenosis/occlusion. However, the often repetitive and stereotyped presentation as well as the temporal clustering around the acute cSAH in symptomatic location suggest that they might be related to the cSAH itself. Due to the potentially irritative nature of cSAH and the repetitive presentation, an epileptic origin is often clinically suspected and antiepileptics are empirically initiated. Though focal or generalised seizures were found in 20–58% of patients in case series with spontaneous cSAHs with aetiologically broader background [11, 29], a cohort study suggested that the cSAH-associated episodes were not attributed to epileptic disturbances, as the EEG did not show epileptic discharges during interictal and (in 1 case) ictal recordings [2]. This is in line with the lack of interictal discharges observed in our patient, and the lack of ictal correlates in another patient with extracranial ICA stenosis-associated cSAH during TFNEs [24]. As a third possibility, it is highly plausible that these transient symptoms are identical to cSAH-related TFNEs seen in CAA, a.k.a. ‘amyloid spells’, where the mechanism is proposed to be cortical spreading depression, similarly to migraine aura [5]. Similarly to cSAH in general, ictal and interictal recordings are generally unrevealing in patients with amyloid spells [5]. The usefulness of antiepileptic medications, including those with anti-migraine properties have been proposed, but systematic data is lacking, and the symptoms might also be self-resolving [5, 30]. In our case, the initiation of antiepileptic medication was followed by abrupt symptomatic relief, with only a single TFNE occurring several weeks later. This episode might be interpreted as one of the following: (1) another spell triggered by a chronic cSAH (i.e., cSS); (2) a TIA-like episode secondary to impaired autoregulation within aberrant cortical/leptomeningeal collateral vasculature during the observed hypertensive surge; (3) a focal aware epileptic seizure arising from prior ischaemic or haemorrhagic left-hemispheric lesions (noting that the patient was on antiepileptic treatment and that all three EEG recordings, one before and two after the event, were normal); (4) a symptomatic presentation of the newly developed left parietal microbleed. Given her young age, the possibility of carotid artery stenting (CAS) was briefly considered, partly for cosmetic reasons. However, the requirement for dual antiplatelet therapy in the context of an apparently haemorrhage-prone collateral vasculature represented a strong contraindication to CAS in this case, and CEA was preferred in accordance with contemporary guidelines. During long-term follow-up, no recurrence of episodes was noted following CEA (staged bilateral in our case), similarly to the few identified prior cases providing follow-up data after CEA [13, 24, 26].

## Conclusion

We conclude that ipsilateral severe stenosis of the extracranial ICA is a rare but clinically relevant proposed cause of cSAH, with a plausible mechanism being the fragility of recruited pial-pial anastomoses. This entity should be considered among the aetiological differential diagnosis of cSAH, and carotid imaging (practically CTA) needs to be incorporated into the diagnostic palette necessary for adequate work-up. On the other hand, cSAH needs to be recognised as a possible manifestation of a severe extracranial ICA stenosis, urging for surgical treatment in addition to best medical therapy. This scenario further enhances the need to carefully look for cSAHs in patients presenting with TFNEs, expanding the potential underlying aetiologies with distinct clinical implications.

## Data Availability

The datasets used and/or analysed during the current study are available from the corresponding author on reasonable request.

## Abbreviations

CAA: Cerebral amyloid angiopathy
CAS: Carotid artery stenting
CEA: Carotid endarterectomy
cSAH: Convexity subarachnoid haemorrhage
cSS: Cortical superficial siderosis
CT: Computed tomography
CVT: Cerebral venous thrombosis
DWI: Diffusion-weighted imaging
EEG: Electroencephalography
ER: Emergency room
FLAIR: Fluid-attenuated inversion recovery
GRE: Gradient echo
ICA: Internal carotid artery
MRI: Magnetic resonance imaging
PRES: Posterior reversible encephalopathy syndrome
RCVS: Reversible cerebral vasoconstriction syndrome
SWI: Susceptibility-weighted imaging
TFNE: Transient focal neurological episode

## References

[1] Khurram A, Kleinig T, Leyden J. Clinical associations and causes of convexity subarachnoid hemorrhage. Stroke. 2014;45(4):1151–3. 24496391 10.1161/STROKEAHA.113.004298

[2] Kumar S, Goddeau RP Jr., Selim MH, Thomas A, Schlaug G, Alhazzani A, et al. Atraumatic convexal subarachnoid hemorrhage: clinical presentation, imaging patterns, and etiologies. Neurology. 2010;74(11):893–9. 20231664 10.1212/WNL.0b013e3181d55efa PMC2836868

[3] Beitzke M, Gattringer T, Enzinger C, Wagner G, Niederkorn K, Fazekas F. Clinical presentation, etiology, and long-term prognosis in patients with nontraumatic convexal subarachnoid hemorrhage. Stroke. 2011;42(11):3055–60. 21921284 10.1161/STROKEAHA.111.621847

[4] Szalardy L, Fakan B, Maszlag-Torok R, Ferencz E, Reisz Z, Radics BL, et al. Identifying diagnostic and prognostic factors in cerebral amyloid angiopathy-related inflammation: A systematic analysis of published and seven new cases. Neuropathol Appl Neurobiol. 2024;50(1):e12946. 38093468 10.1111/nan.12946

[5] Smith EE, Charidimou A, Ayata C, Werring DJ, Greenberg SM. Cerebral Amyloid Angiopathy-Related Transient Focal Neurologic Episodes. Neurology. 2021;97(5):231–8. 34016709 10.1212/WNL.0000000000012234 PMC8356377

[6] Szalardy L, Lee S, Kim A, Kovacs GG. Distinct cerebral amyloid angiopathy patterns in adult Down syndrome. J Neurol Sci. 2025;476:123601. 40639267 10.1016/j.jns.2025.123601

[7] Charidimou A, Linn J, Vernooij MW, Opherk C, Akoudad S, Baron JC, et al. Cortical superficial siderosis: detection and clinical significance in cerebral amyloid angiopathy and related conditions. Brain. 2015;138(Pt 8):2126–39. 26115675 10.1093/brain/awv162

[8] Zedde M, Grisendi I, Assenza F, Napoli M, Moratti C, Pavone C, et al. Spontaneous Non-Aneurysmal Convexity Subarachnoid Hemorrhage: A Scoping Review of Different Etiologies beyond Cerebral Amyloid Angiopathy. J Clin Med. 2024;13:15. 10.3390/jcm13154382 PMC11313189 39124649

[9] Forman R, Conners JJ, Song SY, John S, Garg R, Harris J, et al. The Spectrum of Nontraumatic Convexity Subarachnoid Hemorrhage. J Stroke Cerebrovasc Dis. 2019;28(12):104473. 31677961 10.1016/j.jstrokecerebrovasdis.2019.104473

[10] Aoki N. Do intracranial arteriovenous malformations cause subarachnoid haemorrhage? Review of computed tomography features of ruptured arteriovenous malformations in the acute stage. Acta Neurochir (Wien). 1991;112(3–4):92–5. 1776525 10.1007/BF01405133

[11] Refai D, Botros JA, Strom RG, Derdeyn CP, Sharma A, Zipfel GJ. Spontaneous isolated convexity subarachnoid hemorrhage: presentation, radiological findings, differential diagnosis, and clinical course. J Neurosurg. 2008;109(6):1034–41. 19035716 10.3171/JNS.2008.109.12.1034

[12] Cuvinciuc V, Viguier A, Calviere L, Raposo N, Larrue V, Cognard C, et al. Isolated acute nontraumatic cortical subarachnoid hemorrhage. AJNR Am J Neuroradiol. 2010;31(8):1355–62. 20093311 10.3174/ajnr.A1986 PMC7966116

[13] Geraldes R, Sousa PR, Fonseca AC, Falcao F, Canhao P. Pinho e Melo T. Nontraumatic convexity subarachnoid hemorrhage: different etiologies and outcomes. J Stroke Cerebrovasc Dis. 2014;23(1):e23–30. 24119619 10.1016/j.jstrokecerebrovasdis.2013.08.005

[14] Riley DS, Barber MS, Kienle GS, Aronson JK, von Schoen-Angerer T, Tugwell P, et al. CARE guidelines for case reports: explanation and elaboration document. J Clin Epidemiol. 2017;89:218–35. 28529185 10.1016/j.jclinepi.2017.04.026

[15] Banerjee G, Samra K, Adams ME, Jaunmuktane Z, Parry-Jones AR, Grieve J et al. Iatrogenic cerebral amyloid angiopathy: an emerging clinical phenomenon. J Neurol Neurosurg Psychiatry. 2022;93(7):693–700. 10.1136/jnnp-2022-328792 35577510

[16] Patel KC, Finelli PF. Nonaneurysmal convexity subarachnoid hemorrhage. Neurocrit Care. 2006;4(3):229–33. 16757828 10.1385/NCC:4:3:229

[17] Ly JV, Ma H, Shaloo S, Clissold B, Phan T. Convexity subarachnoid haemorrhage: a practical guide. Pract Neurol. 2023;23(5):368–75. 37116951 10.1136/pn-2022-003572 PMC10579515

[18] Fakan B, Reisz Z, Zadori D, Vecsei L, Klivenyi P, Szalardy L. Predictors of localization, outcome, and etiology of spontaneous intracerebral hemorrhages: focus on cerebral amyloid angiopathy. J Neural Transm (Vienna). 2020;127(6):963–72. 32193732 10.1007/s00702-020-02174-2 PMC7248013

[19] Charidimou A, Boulouis G, Frosch MP, Baron JC, Pasi M, Albucher JF, et al. The Boston criteria version 2.0 for cerebral amyloid angiopathy: a multicentre, retrospective, MRI-neuropathology diagnostic accuracy study. Lancet Neurol. 2022;21(8):714–25. 35841910 10.1016/S1474-4422(22)00208-3 PMC9389452

[20] Gonzalez NR, Amin-Hanjani S, Bang OY, Coffey C, Du R, Fierstra J, et al. Adult Moyamoya Disease and Syndrome: Current Perspectives and Future Directions: A Scientific Statement From the American Heart Association/American Stroke Association. Stroke. 2023;54(10):e465–79. 37609846 10.1161/STR.0000000000000443

[21] Liebeskind DS, Cotsonis GA, Saver JL, Lynn MJ, Cloft HJ, Chimowitz MI. Collateral circulation in symptomatic intracranial atherosclerosis. J Cereb Blood Flow Metab. 2011;31(5):1293–301. 21157476 10.1038/jcbfm.2010.224 PMC3099635

[22] Kuroda S, Fujimura M, Takahashi J, Kataoka H, Ogasawara K, Iwama T, et al. Diagnostic Criteria for Moyamoya Disease – 2021 Revised Version. Neurol Med Chir (Tokyo). 2022;62(7):307–12. 35613882 10.2176/jns-nmc.2022-0072 PMC9357455

[23] Kleinig TJ, Kimber TE, Thompson PD. Convexity subarachnoid haemorrhage associated with bilateral internal carotid artery stenoses. J Neurol. 2009;256(4):669–71. 19401805 10.1007/s00415-009-0106-0

[24] Chandra RV, Leslie-Mazwi TM, Oh D, Mehta B, Yoo AJ. Extracranial internal carotid artery stenosis as a cause of cortical subarachnoid hemorrhage. AJNR Am J Neuroradiol. 2011;32(3):E51–2. author reply E3. 21349955 10.3174/ajnr.A2456 PMC8013084

[25] Geraldes R, Santos C, Canhao P. Atraumatic localized convexity subarachnoid hemorrhage associated with acute carotid artery occlusion. Eur J Neurol. 2011;18(2):e28–9. 20868466 10.1111/j.1468-1331.2010.03221.x

[26] Larrosa D, Ramon C, Benavente L, Calleja S. Convexity subarachnoid haemorrhage secondary to internal carotid stenosis: an indication for revascularisation. BMJ Case Rep. Published online: 26 April 2016. 10.1136/bcr-2016-214661 10.1136/bcr-2016-214661 PMC4854157 27118746

[27] Pereira BJA, de Holanda R, Targino Neto J, de Holanda LF. Spontaneous Convexity Subarachnoid Hemorrhage Caused by Internal Carotid Occlusion: Radiological Features. Arquivos Brasileiros de Neurocirurgia: Brazilian Neurosurg. 2019;39:016–7.

[28] Santamaria-Cadavid M, Rodiguez-Castro E, Lopez-Dequidt I, Arias-Rivas S. Convexity subarachnoid haemorrhage associated with ipsilateral carotid artery occlusion. Neurología (English Edition). 2020;35(7):538–40. 10.1016/j.nrl.2018.09.002 30442445

[29] Spitzer C, Mull M, Rohde V, Kosinski CM. Non-traumatic cortical subarachnoid haemorrhage: diagnostic work-up and aetiological background. Neuroradiology. 2005;47(7):525–31. 15971064 10.1007/s00234-005-1384-6

[30] Charidimou A, Baron JC, Werring DJ. Cerebral amyloid angiopathy and transient focal neurological episodes. Cerebrovasc Dis. 2013;36(3):245–6. 24135538 10.1159/000353989

